# Fatal *Sarcocystis falcatula* Infection in Three Penguins

**DOI:** 10.3389/fvets.2019.00340

**Published:** 2019-10-10

**Authors:** Shannon G. M. Kirejczyk, Rachel E. Burns, Michael W. Hyatt, Michael J. Yabsley, Julia M. Ter Beest, Zoltan S. Gyimesi, Robert J. Ossiboff, Amelia Waltman, Tracie A. Seimon, Rita McManamon

**Affiliations:** ^1^Department of Pathology, College of Veterinary Medicine, University of Georgia, Athens, GA, United States; ^2^Connecticut Veterinary Medical Diagnostic Laboratory, Department of Pathobiology and Veterinary Science, College of Agriculture, Health and Natural Resources, University of Connecticut, Storrs, CT, United States; ^3^Adventure Aquarium, Camden, NJ, United States; ^4^Southeastern Cooperative Wildlife Disease Study, College of Veterinary Medicine, University of Georgia, Athens, GA, United States; ^5^Warnell School of Forestry and Natural Resources, University of Georgia, Athens, GA, United States; ^6^Louisville Zoological Garden, Louisville, KY, United States; ^7^The Wildlife Conservation Society, Bronx, NY, United States; ^8^Zoo and Exotic Animal Pathology Service and Infectious Diseases Laboratory, Department of Small Animal Medicine and Surgery, College of Veterinary Medicine, University of Georgia, Athens, GA, United States

**Keywords:** penguin, protozoal pneumonia, *Sarcocystis falcatula*, apicomplexa, *Spheniscus demersus*, *Eudyptes chrysocome*

## Abstract

*Sarcocystis falcatula* is a well-known cause of fatal pneumonia in some birds, particularly Old World psittacines. Here we describe fatal sarcosystosis due to *S. falcatula* in 3 penguins (Family Spheniscidae) under managed care, including one African penguin (*Spheniscus demersus*), and two Southern rockhopper penguins (*Eudyptes chrysocome*). Randomly distributed foci of necrosis, inflammatory cell infiltrates, edema, and variable numbers of round to elongated protozoal schizonts were observed in sections of lung. Protozoal organisms exhibited strong immunoreactivity for *Sarcocystis* sp. antigen by immunohistochemistry. Apicomplexan and *Sarcocystis* genus-specific PCR assays and sequence analysis confirmed *S. falcatula* as the etiologic agent. These cases of fatal pneumonia attributed to *S. falcatula* expand the list of aberrant intermediate avian hosts, with particular implications for penguins.

## Background

Causes of protozoal pneumonia in birds include *Sarcocystis falcatula, Toxoplasma gondii, Plasmodium* spp., *Haemoproteus* spp., and *Isospora* (formerly *Atoxoplasma*) spp. Such infections are characterized by necrotizing interstitial pneumonia, with air capillaries containing tachyzoites (*T. gondii*) or meronts/schizonts (*S. falcatula* and *Plasmodium* spp.) within endothelial cells and/or macrophages. Malaria and toxoplasmosis are well-described causes of interstitial pneumonia in penguins (1, 2). Although molecular evidence of *Sarcocystis* spp. infection was recently reported in Magellenic penguins in Brazil (3), fatal disease due to pulmonary sarcocystosis has not previously been described in penguins. In this case series, we report the clinical, gross, microscopic, and ultrastructural features of fatal *S. falcatula* infections in 3 penguins under managed care.

## Case Presentations

Case 1 was a 27-year-old, male African penguin (*Spheniscus demersus*) housed at an aquarium in the United States. The bird was at the end of a molting period and had been depressed and lethargic for a couple days before being found dead. Necropsy revealed congested lungs, and a complete set of formalin-fixed tissues was submitted to the Connecticut Veterinary Medical Diagnostic Laboratory for histopathology. Opossums had been seen on the property where this penguin was housed, but not inside the exhibit.

**Figure 1:**
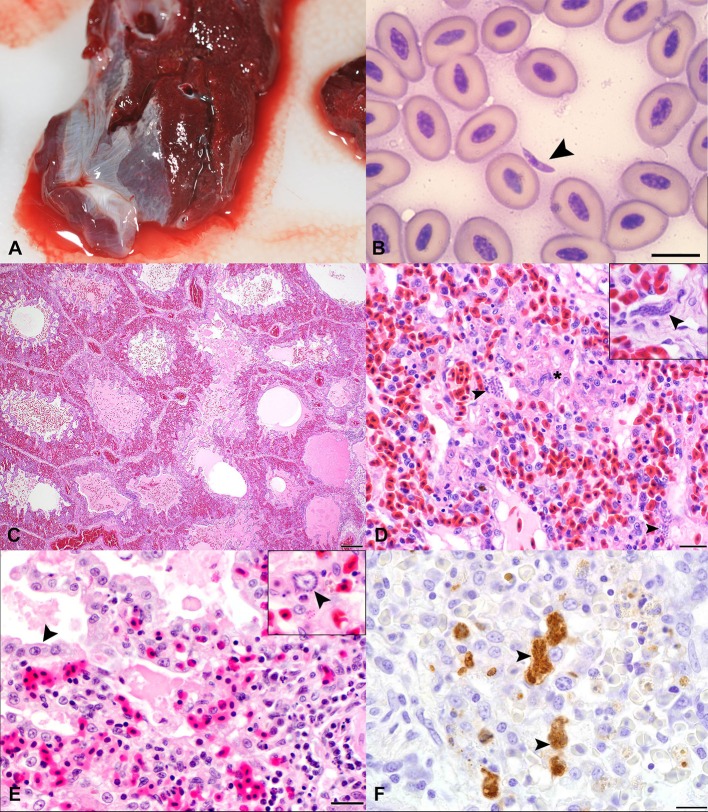
Gross, cytological, histopathologic, and immunohistochemical features of *Sarcocystis falcatula* infection in the lung of Southern rockhopper (*Eudyptes chrysocome*) and African (*Spheniscus demersus*) penguins. **(A)** Gross appearance of lung tissue from case 2 at necropsy. The lungs are dark red and edematous, with red-tinged fluid oozing along the periphery of the tissue. The lungs sank in formalin. **(B)** Cytology of tissue imprint from the lung of case 3 obtained at necropsy. The vast majority of cells are erythrocytes. A single, 2 × 8 micron, elongate to crescent-shaped tachyzoite with a small, round, slightly off-center nucleus is present at the center of the image (arrowhead). Bar = 10 μm. **(C)** Lung, from a 5-year-old, female, Southern rockhopper penguin (case 2), with *S. falcatula* pneumonia. Parabronchi and atria are frequently flooded by edema, hemorrhage and fibrin. H&E. Bar = 200 μm. **(D)** Lung, case 2. Air capillaries are multifocally obscured by foci of necrosis (^*^) containing protozoal schizonts (arrowheads), fibrin, hemorrhage, and low numbers of heterophils. The interstitium is hypercellular due to low numbers of lymphocytes and macrophages. H&E. Bar = 20 μm. Inset: High magnification image highlights the elongate to serpentine appearance of an intracellular schizont (case 3). **(E)** Lung, case 1. Pneumocytes lining the parabronchus in the top left of the image are hypertrophied (arrowhead) and the air space is filled with edema, fibrin, macrophages, and scant hemorrhage. The interstitium is expanded by inflammatory cell infiltrates, and multifocally obscured by necrosis, hemorrhage, and an accumulation of fibrin and edema. H&E. Bar = 20 μm. Inset: High magnification image with a schizont (arrowhead), which exhibits a “sunburst” pattern, with merozoites radiating around a clear zone. **(F)** Immunohistochemistry for *S. neurona* polyclonal antibody on lung tissue (case 2) demonstrates strongly immunoreactive, elongate to serpentine schizonts (arrowheads), free merozoites, and macrophages containing phagocytosed debris. DAB chromogen with hematoxylin counterstain. Bar = 10 μm.

Case 2 was a 5-year-old, female, Southern rockhopper penguin (*Eudyptes chrysocome*) from a different zoological institution. Due to renovation of the birds' indoor exhibit, the penguin and its flock were temporarily housed outdoors for 6 weeks in a completely meshed enclosure with access to a temperature-regulated pool. Penguins were given oral itraconazole^1^ (15 mg, once daily) for aspergillosis prophylaxis for the duration of the relocation. Two days prior to its death, the bird's appetite declined and it was observed floating in water, rather than exhibiting normal swimming and diving behavior. Physical examination revealed severe dyspnea and generalized weakness. The bird was anesthetized for further diagnostics but died shortly after induction. Post-mortem radiographs revealed increased soft tissue opacity in the lungs. Necropsy revealed dark red, wet lungs that sank in formalin (Figure 1A), a friable spleen, and edematous pericardial sac. There was a well-demarcated, 1-cm-diameter, white to yellow, raised, coelomic plaque on the inner surface of the ribs and a moderate amount of green fecal staining around the cloacal orifice. Following the death of this bird and the onset of warmer spring temperatures, the remaining flock was moved to an indoor, chilled holding area.

Five days later, a 32-year-old, female, Southern rockhopper penguin (case 3) from the same institution began to exhibit weakness, anorexia and dyspnea, and auscultation revealed harsh lung sounds. The bird was treated with ponazuril^2^ (25 mg/kg PO, once), enrofloxacin^3^ (15 mg/kg SC, once), meloxicam^4^ (0.5 mg/kg IM, once), and furosemide^5^ (0.2 mg/kg IM, once) for presumed pulmonary edema, but died 1 h later. Necropsy examination revealed dark red, wet lungs, and an enlarged, nodular spleen. The caudal aspect of the left lung contained a small, focal, tan to brown, firm area.

Cytologic examination of lung tissue imprints from case 3 showed crescent-shaped, 2 × 4–8 μm protozoal zoites (Figure 1B), toxic heterophils and multinucleated giant cells. A complete set of tissues from cases 2 and 3 were fixed in 10% neutral-buffered formalin and submitted to the University of Georgia Zoo and Exotic Animal Pathology Service for histopathology. Fresh spleen from case 2 was submitted to Athens Veterinary Diagnostic Laboratory for aerobic and anaerobic cultures. Fresh lung and liver from Cases 2 and 3 were submitted to the Southeastern Cooperative Wildlife Disease Study (SCWDS, Athens, GA) for molecular testing.

## Laboratory Investigations and Diagnostic Tests

### Histopathology and Immunohistochemistry

Representative sections of all submitted tissues were routinely processed, embedded in paraffin wax, and 4–5-micron-thick sections were stained with Hematoxylin and Eosin (H&E) for light microscopy. For case 1, the only relevant immunohistochemical stain available at the Connecticut Veterinary Medical Diagnostic Laboratory was a rabbit polyclonal antibody against *T. gondii* (BioGenex, San Ramon, CA). Additional stains for *Sarcocystis* sp. (rabbit polyclonal antibody) and *T. gondii* (rabbit polyclonal antibody) were performed at the California Animal Health & Food Safety Laboratory System, Davis, CA as previously described (4). For cases 2 and 3, immunohistochemistry was performed at the University of Georgia College of Veterinary Medicine Histology Laboratory using antibodies for *S. neurona* (rabbit polyclonal antibody, 1:500 dilution for 60 min)*, Neospora caninum* (goat polyclonal antibody^6^, 1:300 dilution for 30 min) and *T. gondii* (mouse monoclonal antibody^6^, 1:1,0000 dilution for 10 min).

The clinical, histologic, immunohistochemical, and molecular findings for three penguins with fatal *S. falcatula* infection are summarized in Table 1. All penguins had severe, necrotizing and lymphohistiocytic interstitial pneumonia. Parabronchi were flooded with hemorrhage, edema, and fibrin (Figure 1C). Air capillaries were obscured by foci of necrosis with fibrin exudation, and accumulations of heterophils, hemorrhage, and protozoal schizonts (Figure 1D). Air spaces were multifocally lined by hypertrophied epithelial cells (Figure 1E). In cases 2 and 3, schizonts were numerous and elongate, often conforming to the shape of capillaries (Figure 1D, inset), while schizonts were fewer and this classic serpentine morphology of *S. falcatula* was not observed in case 1. In all cases, schizonts occasionally exhibited a “sunburst” arrangement, in which merozoites radiated around a centralized clearing (Figure 1E, inset).

**Table 1:** Signalment, clinical history, gross pathology, histopathology, immunohistochemistry, and molecular findings for 3 penguins with *S. falcatula* infection.

**Case**	**Signalment**	**Clinical history and gross pathology**	**Histopathology**	**Immunohistochemistry**			**PCR results**	
				***Sarcocystis***	***Neospora***	***Toxoplasma***	***Sarcocystis***	***Plasmodium***
1	27-year-old, male African penguin (*Spheniscus demersus)*	Several days of depression and lethargy; found dead Congested lungs	^*^Lymphohistiocytic and necrotizing pneumonia with protozoal schizonts Lymphohistiocytic hepatitis Pulmonary anthracosis	+++	ND	±	*S. falcatula*	ND
2	5-year-old, female, Southern rockhopper penguin (*Eudyptes chrysocome*)	Housed outdoors for 6 weeks in meshed enclosure; access to temperature-regulated pool; itraconazole^1^ prophylaxis (9 mg/kg) Anorexia, abnormal swimming, and severe dyspnea; died while anesthetized for exam Dark red, wet lungs Friable spleen	^*^Necrotizing pneumonia with protozoal schizonts Splenic necrosis Sepsis (*Clostridium perfringens* cultured from spleen). Lymphohistiocytic myocarditis Lymphohistiocytic myositis Lymphohistiocytic hepatitis Lymphohistiocytic ventriculitis Extramedullary granulopoiesis in liver and spleen Schizonts in lung, heart, skeletal muscle, liver, proventriculus, ventriculus, duodenum, pancreas, adrenal, and kidney Small fungal granuloma (coelomic plaque)	+++	ND	ND	*S. falcatula*	–
3	32-year-old, female, Southern rockhopper penguin (*Eudyptes chrysocome*)	Housed outdoors for 6 weeks in meshed enclosure; access to temperature-regulated pool; itraconazole prophylaxis (6.5 mg/kg) Anorexia and severe dyspnea; given ponazuril (25 mg/kg PO), enrofloxacin (15 mg/kg SC), meloxicam (0.5 cm/kg IM), and furosemide (0.2 mg/kg IM); died 1 h post-exam Dark red, wet lungs Spleen enlarged, mottled, and nodular	^*^Necrotizing pneumonia with protozoal schizonts Splenic necrosis Lymphohistiocytic myocarditis Lymphohistiocytic myositis Lymphohistiocytic hepatitis Koilin degeneration Extramedullary granulopoiesis in liver and spleen Schizonts in lung, heart, skeletal muscle, spleen, liver, brain, and kidney Pulmonary granuloma and air sacculitis	+++	+	–	*S. falcatula*	–

* *Presumptive cause of death; –, Immunonegative; +, Weakly immunopositive; +++, Strongly immunopositive; ND, not done*.

A full set of tissues, including lung, liver, brain, skeletal muscle, and heart was examined for case 1. No extrapulmonary schizonts or sarcocysts were seen, and additional immunohistochemical stains were not pursued. Cases 2 and 3 exhibited mild lymphohistiocytic myocarditis and myositis and schizonts were observed in multiple tissues in these birds (Table 1). Protozoa stained variably PAS-positive on Periodic acid-Schiff reaction stains, and did not stain with Giemsa. On immunohistochemistry, protozoa exhibited strong immunoreactivity (Figure 1F) for polyclonal *S. neurona* antibodies and variable immunoreactivity for *N. caninum* and *T. gondii* antibodies (Table 1).

**Figure 2:**
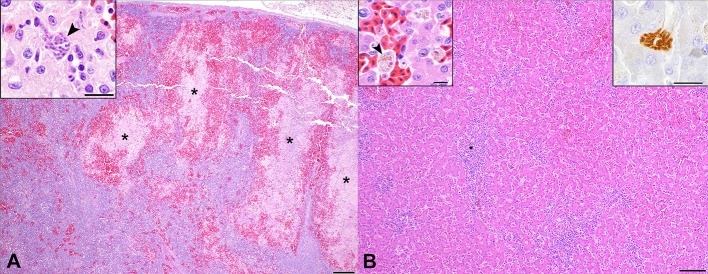
Hepatic and splenic pathology in Southern rockhopper penguins with fatal *Sarcocystis falcatula* infection. **(A)** Spleen, case 2. The splenic parenchyma is markedly hypercellular, with numerous hematopoietic cell precursors, lymphocytes, histiocytes, and plasma cells. There are multifocal to coalescing zones of coagulative to lytic necrosis (*) surrounded by a rim of hemorrhage. H&E. Bar = 200 μm. Inset: High magnification image of a *S. falcatula* schizont (arrowhead) within an area of necrosis in the spleen of case 3. Bar = 10 μm. **(B)** Liver, case 2. Portal regions are hypercellular, with moderate numbers of lymphocytes, plasma cells, macrophages and granulocytic precursor cells, which compress hepatocytes multifocally. Bar = 100 μm. Left insert: Sinusoids are congested, with macrophages containing phagocytosed cell debris, erythrocyte fragments and hemosiderin (arrowhead). H&E. Bar = 10 μm. Right inset: Immunohistochemistry for *S. neurona* highlights a schizont with radially arranged merozoites. Bar = 10 μm.

Cases 2 and 3 had markedly hypercellular spleens with foci of extramedullary granulopoiesis. There were also coalescing foci of coagulative to lytic necrosis, and case 3 had scattered, intraendothelial schizonts (Figure 2A). Under anaerobic conditions, *Clostridium perfringens* was cultured from the spleen of case 2. Splenic tissue from case 3 was not available for culture. Low numbers of schizonts were present in foci of splenic necrosis for case 3, so sarcosporidiosis is the most likely explanation for this lesion. However, given that low numbers of gram-positive bacilli in one section of skeletal muscle, septicemia cannot entirely be ruled out in case 3.

All cases had multifocal lymphohistiocytic portal hepatitis and extramedullary granulopoiesis (Figure 2B); cases 2 and 3 had low numbers of schizonts within sinusoids (Figure 2B, right inset). All cases also had moderate numbers of sinusoidal macrophages containing fragments of erythrocytes and intracytoplasmic hemosiderin (Figure 2B, left inset).

In addition to fulminant pulmonary sarcocystosis, case 2 had a small, coelomic fungal granuloma. Aspergillosis is suspected but fungal culture and/or PCR would be required for definitive diagnosis and further testing was not pursued. Case 3 had mild air sacculitis and a focal heterophilic granuloma at the caudal aspect of the left lung lobe. This lesion is believed to be related to a prior aspiration event, given the presence of foreign material and lack of microorganisms on special stains for fungi (Gomori Methenamine Silver), bacteria (modified Brown and Brenn Gram), and acid-fast bacilli (Ziehl-Neelsen).

### Transmission Electron Microscopy

Formalin-fixed lung tissue from case 3 was trimmed into two, 2-mm-thick pieces and transferred to 2% paraformaldehyde, 2% glutaraldehyde in 0.1 M phosphate buffer, pH 7.25. After overnight fixation, tissue was rinsed in 0.1 M phosphate buffer, post-fixed for 1 h with 1% buffered osmium tetroxide (OsO_4_), then rinsed in deionized water and dehydrated in an ascending ethanol series before infiltration with propylene oxide and Mollenhauer's Epon-Araldite resin mixture (5). Tissue samples were embedded in fresh resin mixture and allowed to polymerize in a 70°C oven. One-micron-thick sections were stained with Toluidine Blue O to select areas of interest before 60 nm sections were obtained and placed on grids. Grid sections were stained with uranyl acetate and lead citrate before examination with a JEOL JEM 1011 transmission electron microscope at 80 kV. Images were captured with an XR80M wide-angle multi-discipline mid-mount CCD camera^7^.

**Figure 3:**
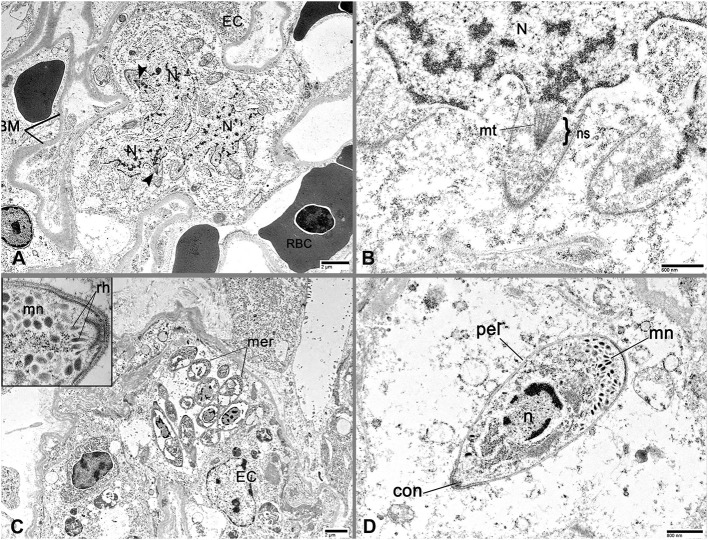
Ultrastructure of *Sarcocystis falcatula* asexual life stages by Transmission Electron Microscopy (TEM) in the lung of a 32-year-old, female, Southern rockhopper penguin (case 3). **(A)** TEM demonstrates an intraendothelial *S. falcatula* schizont. The schizont expands the endothelial cell (EC), is irregularly shaped and is separated from the EC cytoplasm by a thin membrane. Several nuclear spindles (arrowheads) project from the schizont nucleus (N). The endothelial cell abuts a capillary containing erythrocytes (RBC) and is supported by a basement membrane (BM). Bar = 2 μm. **(B)** Higher magnification TEM image of the *S. falcatula* schizont nucleus (N) from **(A)**. Several conically arranged microtubules (mt) comprise the nuclear spindle (ns) of a developing merozoite. Bar = 600 nm. **(C)** TEM shows numerous, up to 2 × 6 μm merozoites clustered within an endothelial cell (EC). Inset: Higher magnification of a merozoite showing rhoptries (rh) and micronemes (mn). Bar = 2 μm. **(D)** A mature merozoite is present in a focus of necrotic lung tissue on TEM. The merozoite has a central, round nucleus (n), an anterior conoid (con), numerous micronemes (mn) at the posterior pole, and a double-membrane pellicle (pel). Bar = 800 nm.

Transmission electron microscopy revealed protozoa with ultrastructural features compatible with *Sarcocystis* spp. (Figure 3) (6–8). Capillary endothelial cells occasionally contained an intracytoplasmic, serpentine schizont, which conformed to the cell shape. One schizont had a multilobulated nucleus with nuclear spindles arranged along the periphery (Figures 3A,B). Merozoites had a round, central nucleus, an anterior conoid and several tear-shaped, electron-dense micronemes at the posterior end (Figures 3C,D) (6).

### Polymerase Chain Reaction (PCR) Analysis and Sequencing

For case 1, formalin-fixed, paraffin-embedded (FFPE) lung tissue was submitted to the Wildlife Conservation Society (Bronx, NY) and DNA was extracted using a QIAamp DNA FFPE Tissue Kit^8^. Extracts were tested by conventional PCR using both pan-apicomplexan and *Sarcocystis*-specific assays targeting the 18S rRNA and internal transcribed spacer 1 (ITS-1) regions, respectively. A small portion of the 18S rRNA gene was amplified as previously described (9). A portion of the ITS-1 region was amplified using primer P-ITSF and a degenerate primer (ShortITSR; 5′-GGGATTCARTKGYYGAAA-3′) designed based on publicly available Sarcocystis ITS-1 sequences (10). Amplicons were bi-directionally sequenced commercially, analyzed using Geneious R7 (Auckland, New Zealand) to generate a consensus sequence, trimmed of their primers, and analyzed by BLASTn.

The DNA sequence from the 18S gene was 100% identical to *Sarcocystis falcatula* in Genbank (MH626537, isolate Lorikeet ID #205850) but also identical to numerous other *Sarcocystis* isolates in GenBank. The DNA sequence from the ITS-1 region was 100% identical to *Sarcocystis falcatula* in GenBank (MH626538, isolate Lorikeet ID #205850) and *Sarcocystis cf. falcatula* in GenBank (AF389339) (11, 12). The next closest match for the ITS-1 region was *Sarcocystis speeri* and *Sarcocystis neurona* (98–99% identity).

For cases 2 and 3, DNA was extracted from fresh lung and liver using a commercial DNA extraction kit^8^ at SCWDS. The genus of the organism was determined by screening tissues with primers that amplify a short portion of the 18S rRNA gene of numerous apicomplexan parasites as previously described (13). Amplification products were visualized in 2% agarose gels stained with GelRed^9^. Amplicons were gel-purified using a kit^8^ and bi-directionally sequenced at the University of Georgia Genomics Facility (Athens, Georgia). Chromatograms were analyzed using Geneious R7 and the consensus sequence was compared to other sequences in GenBank.

To further characterize the *Sarcocystis* sp. in cases 2 and 3, partial cytochrome b gene was amplified with primers CYTB-F and CYTB-R, the ITS-1 region was amplified with primers ITS-234F19 and ITS-720R19, and the partial surface antigen 2 gene was amplified with primers SAG2-F1 and SAG2-R1 as previously described (14, 15). Amplicons were purified, sequenced, and analyzed as described above. To rule out the possibility of *Plasmodium* and/or *Haemoproteus* spp. infection in cases 2 and 3, a nested PCR targeting the mitochondrial cytochrome b (*cytb*) gene was conducted as previously described using primary primers HaemNFI and HaemNR3 and nested primers HaemF and HaemR2 (16).

For cases 2 and 3, liver and lung samples from both penguins were positive using the Tg18s58F and Tg18s348R PCR protocol and the resulting sequences (302 bp) were identical to each other and 100% similar to numerous *Sarcocystis* spp. The cytb gene sequences (580 bp) obtained from liver and spleen of cases 2 and 3 were identical and 100% similar to *S. falcatula* from captive bare-faced ibis (*Phimosus infuscatus*) from Brazil (KX265018) and Virginia opossum from California, USA (KP871704). The ITS-1 (287 bp) sequences were identical to each other and contained two polymorphic bases. These sequences were 98–99% similar to numerous *S. falcatula* strains from Brazil and the U.S.A. with some only differing at those two polymorphic bases. The four SAG2 sequences (402 bp) were identical and were 100% similar to the only *S. falcatula* (GQ851953) sequence available in GenBank. All four tissues were positive for the rpoB gene. The sequences were identical to each other and 100% similar to one *S. falcatula* (440 bp, KX265017) strain and >99% similar to several other *S. falcatula* sequences in GenBank (495 bp, e.g., AY164999).

## Discussion

Death of these penguins was attributed to severe pneumonia caused by *S. falcatula*. The diagnosis was supported by IHC and confirmed by PCR. The differential diagnosis for apicomplexan pneumonia in penguins includes malaria, toxoplasmosis, and sarcocystosis. Fatal pulmonary infection with *S. falcatula* has been reported most often in psittacines (17–20), and the current report is believed to be the first in Spheniscidae. The histopathologic features of pneumonia in these penguins were similar to that described in other avian species infected with *S. falcatula*, namely necrosis, edema, fibrin deposition, congestion, hemorrhage, heterophilic, and mononuclear inflammatory infiltrates, endothelial cell lysis, and pneumocyte hyperplasia (7, 20–22).

The life cycle of *Sarcocystis* spp. involves definitive and intermediate hosts. In North America, the definitive host for *S. falcatula* and the closely related *S. neurona* is the Virginia opossum (*Didelphis virginiana*), which sheds infective sporocysts in feces (8). Infection of intermediate hosts typically occurs through ingestion of food contaminated with opossum feces, but insects can serve as mechanical vectors (17). In case 1, opossums were seen on the premises but not in the bird's enclosure, so ingestion of feces or sporozoite-containing fomite(s) is considered most likely. Contaminated water run-off or insect entry into the enclosure are additional possibilities.

In contrast to case 1 (African penguin), cases 2 and 3 (Southern rockhopper penguins) had disseminated infections with large numbers of schizonts. Potential factors that may have influenced the progression of disease in these animals include the dose of sporozoites ingested, time elapsed since infection, differences in host species susceptibility or pathogen virulence, and concomitant sepsis in case 2.

Acosta et al. (3) recently provided molecular evidence of *Sarcocystis* spp. infection in a cohort of Magellanic penguins (*Spheniscus magellanicus*) in Brazil. A *Sarcocystis* sp. closely related to *S. falcatula* was isolated from the pectoral muscle of 16 penguins, which were undergoing rehabilitation and died due to other causes. Given the tissue of origin (pectoral muscle), the authors postulated that the genetic material originated from tissue cysts, suggesting that the penguins survived an acute infection and formed sarcocysts. Sarcocysts were not identified in the penguins of the current report.

Cross-reactivity between cyst-forming apicomplexans has been reported for polyclonal antibodies targeting *T. gondii, N. caninum*, and *Sarcocystis* sp. (23–25). Although a previous study has shown a lack of cross-reactivity between a polyclonal *S. neurona* antibody and *S. falcatula* schizonts in budgerigars (*Melopsittacus undulatus*) (26), the *S. neurona* antibody used in all cases strongly reacted with *S. falcatula* in the tissue sections, which highlights the variability that can occur between IHC results based on different polyclonal antibodies.

This report expands the list of intermediate hosts for *S. falcatula* and underscores the importance of excluding opossums, their feces, and potential vectors from penguin enclosures. Additional studies are needed to determine individual and species-specific susceptibilities among penguins and whether or not infection and survival confers protective humoral immunity in individual birds. Prophylactic treatment for surviving penguins may be considered if sarcocystosis is diagnosed in a collection.

## Data Availability Statement

All datasets generated for this study are included in the manuscript/supplementary files.

## Ethics Statement

All animals included in this study were treated with the standard of care at each institution, as set forth by the Association of Zoos and Aquariums. An ethical review process was not required for this study, as the methods described herein were part of the routine diagnostic process to determine the cause of death of these penguins. All biological samples were obtained from penguins at postmortem exam, following the natural death of these animals.

## Author Contributions

SK and RB drafted the manuscript. MH provided clinical care for case 1 and performed the post mortem exam. JT and ZG provided clinical care for cases 2 and 3 and performed the post mortem exams. RB performed the histopathology on case 1. SK and RM performed the histopathology on cases 2 and 3 and TEM on case 3. RO, TS, and AW designed and performed the PCR and sequencing and analyzed the sequencing data for case 1. MY designed and performed the PCR and sequencing and analyzed the sequencing data for cases 2 and 3. All authors assisted with writing and critically reviewing the manuscript.

### Conflict of Interest

The authors declare that the research was conducted in the absence of any commercial or financial relationships that could be construed as a potential conflict of interest.

## Footnotes

^1^Janssen Ortho LLC, Stateroad 933 Km 0 1 Street Statero, Gurabo, Puerto Rico 00778 USA. 100 mg capsules were opened and weighed out into 15 mg capsule doses by JT or ZG or by a compounding pharmacy (Taylors Pharmacy, 306 South Park Avenue, Winter Park, FL 32789 USA).

^2^Compounded by Taylors Pharmacy, 306 South Park Avenue, Winter Park, FL 32789 USA. 50 mg/ml suspension.

^3^Manufactured by Norbrook Laboratories Limited, Newry, BT35 6PU, Co. Down, Northern Ireland. 22.7 mg/ml.

^4^Manufactured by Norbrook Laboratories Limited, Newry, BT35 6PU, Co. Down, Northern Ireland. 5 mg/ml.

^5^Manufactured by Boehringer Ingelheim Vetmedica, Inc. 2621 North Belt Hwy, St. Joseph, MO 64506, USA.

^6^Veterinary Medical Research & Development, 425 NW Albion Dr, Pullman, WA, 99163, U.S.A.

^7^Advanced Microscopy Techniques, 242 West Cummings Park, Woburn, MA, 01801, U.S.A.

^8^QIAGEN, Inc, 27220 Turnberry Lane 200, Valencia, CA 91355, U.S.A.

^9^Biotium, Inc. 46117 Landing Pkwy, Fremont, CA 94538, U.S.A.
